# Smartphone Use and Postural Balance in Healthy Young Adults

**DOI:** 10.3390/ijerph17093307

**Published:** 2020-05-09

**Authors:** Roxana Ramona Onofrei, Elena Amaricai, Oana Suciu, Vlad Laurentiu David, Andreea Luciana Rata, Elena Hogea

**Affiliations:** 1 Department of Rehabilitation, Physical Medicine and Rheumatology, “Victor Babes” University of Medicine and Pharmacy Timisoara, 300041 Timisoara, Romania; onofrei.roxana@umft.ro (R.R.O.); amaricai.elena@umft.ro (E.A.); oanasuciu78@umft.ro (O.S.); 2 Department of Pediatric Surgery and Orthopedics, “Victor Babes” University of Medicine and Pharmacy Timisoara, 300041 Timisoara, Romania; david.vlad@umft.ro; 3 Department of Vascular Surgery, “Victor Babes” University of Medicine and Pharmacy Timisoara, 300041 Timisoara, Romania; 4 Department of Microbiology, “Victor Babes” University of Medicine and Pharmacy Timisoara, 300041 Timisoara, Romania; hogea.elena@umft.ro

**Keywords:** postural stability, smartphone, talking, texting

## Abstract

Maintaining an upright posture while talking or texting on the phone is a frequent dual-task demand. Using a within-subjects design, the aim of the present study was to assess the impact of a smartphone conversation or message texting on standing plantar pressure and postural balance performance in healthy young adults. Thirty-five subjects (mean age 21.37 ± 1.11 years) were included in this study. Simultaneous foot plantar pressure and stabilometric analysis were performed using the PoData system, under three conditions: no phone (*control*), talking on a smartphone (*talk*) and texting and sending a text message via a smartphone (*text*). Stabilometric parameters (center of pressure (CoP) path length, 90% confidence area and maximum CoP speed) were significantly affected by the use of different smartphone functions (*p* < 0.0001). The CoP path length and maximum CoP speed were significantly higher under the *talk* and *text* conditions when compared to the *control*. CoP path length, 90% confidence area and maximum CoP speed were significantly increased in *talk* compared to *text* and *control*. Talking on the phone also influenced the weight distribution on the left foot first metatarsal head and heel as compared with message texting. Postural stability in healthy young adults was significantly affected by talking and texting on a smartphone. Talking on the phone proved to be more challenging.

## 1. Introduction

Maintaining an upright posture while executing a concurrent task represents a common daily activity. This implies dual-task performance. It has been shown that the attentional requirements of a concurrent cognitive task have an impact on postural stability, as quantified by the stability of the center of pressure or the measurement of the sway path [1,2,3,4]. The effects of a concurrent task on postural control are controversial, depending on the task, age of the tested subjects or associated pathologies. While some studies noted less postural sway when concurrently performing a cognitive task (counting backwards, mathematical addition problem) in healthy young adults [5,6,7], the detrimental effect of a secondary visuospatial attention-demanding task on postural control was observed in middle-aged and older adults [8,9]. Talking has also been proved to have an impact on postural balance that could be attributed to the respiratory muscle activity related to vocalization [10]. Yardley et al. reported an increased instability during a spoken task, but not during a silent one, that was mainly due to the effects of articulation rather than to competing demands for attention. Articulation, mediated by the respiratory activity involved in speech, significantly increased the sway path [11]. Dault et al. also noted that the articulation needed to execute the spoken tasks could have provoked respiratory changes, leading to a pronounced increase in the postural sway [12].

Since advances in smartphone technology have transformed this hand-held device into an everyday social and/or professional necessity, maintaining an upright posture or walking while talking or texting on the phone are frequently met dual-task demands. The safe use of a phone during daily activities, like standing, walking, stair gait, crossing streets and driving has been studied, since all these acts require cognitive and motor abilities, as well as the division of attention [13,14,15,16,17,18,19]. Texting proved to have a higher detrimental effect on balance, gait and walking behavior in comparison to talking on the phone, increasing the postural sway and decreasing the gait velocity, stride length and cadence [4,18,20]. Nurwulan et al. found that texting affected the postural balance both in normal and tandem stance in healthy college students [14]. Strubhar et al. also reported that cell phone texting had an impact both on the reactive balance and on the gait. Their results showed that healthy individuals had more difficulty controlling their center of mass during perturbed stance while texting. This finding may be due to an interference with the participant’s orientation to the vertical [21]. The standing balance in healthy young individuals was also affected by texting in the study of Goddard et al. [22]. Cho et al. reported a decrease in dynamic balance ability during smart phone texting in healthy young individuals [13].

Smartphone usage was also found to have a detrimental effect on posture, especially on the neck and trunk regions [23,24,25]. These changes could influence the plantar pression distribution and also the stabilographic variables [26]. Plantar pressure measurements in bipedal stance is a useful tool to assess both the foot loading and the posture [27].

To the best of our knowledge, there are no studies that have investigated the effects of talking and texting on a smartphone upon postural stability and plantar pressure. Therefore, based on the previous studies that showed the detrimental effect of phone use, especially texting, on balance and gait, we hypothesized that (1) the cognitive and motor demands of using a smartphone (conversation and message texting) will modify the standing plantar pressure and will also have an effect on the postural stability and (2) the message texting dual-task will be more demanding than talking. Using a within-subjects design, the aim of the present study was to assess the impact of smartphone conversations and message texting on standing plantar pressure and in postural balance performance in healthy young adults.

## 2. Materials and Methods

### 2.1. Participants

Healthy young subjects were asked to participate in the study. They were recruited among the graduates of our university, their friends, relatives, or acquaintances. Subjects with vestibular or visual problems, neurological or musculoskeletal disorders that would affect their postural balance were not included. Participation in the study was voluntary. Written informed consent was obtained from all the participants, as an instrument for proper information processing, improvement of decision making capacity, data base collection and processing [28]. The sample size was calculated using G*Power software version 3.1.9.2 (Christian-Albrechts-Universität Kiel, Kiel, Germany). For α = 0.05, β = 0.2, a medium effect size (f = 0.25), with three measurements, a sample size of at least 28 subjects would be required [29].

Thirty-five subjects (mean age 21.37 ± 1.11 years) were included in this study. The demographic characteristics are presented in Table 1. All the subjects were right-handed.

The study protocol was explained to all subjects. The study has been carried out in accordance with the Declaration of Helsinki and was approved by the Institutional Ethics Committee (Victor Babes University of Medicine and Pharmacy Timisoara, No. 23/2019).

### 2.2. Assessments

Anthropometric data were collected for all the participants (age, gender, weight, height). Simultaneous static plantar pressure and stabilometric analysis were performed using the PoData system (Chinesport, Udine, Italy), a capacitive pressure distribution system with an integrated podoscope and six load cells, connected to a computer with the GPS5 software, with a sampling frequency of 100 Hz. Each of the two thick nondeformable glass platforms rests on three points corresponding to three load cells, forming an isosceles triangle. Each cell is measuring the force on one point, having the following characteristics: dimensions Ø 35 mm, h 12 mm, maximal load 100 kg, with a calibration tolerance of ±0.2% full scale (FS), linearity, hysteresis and repeatability of ±0.2% FS, temperature effect ±0.03% FS, accuracy worst case 1% FS. The podoscope allows for the acquisition of a webcam photograph of the way the foot interacts with the supporting surface. The software identifies via the webcam the position of the six load cells and also allows the investigator to position the virtual sensors on the podoscopic image on main contact points (1st and 5th metatarsal heads and the heels), based on the studies of Kapandji [30]. The system provides information about weight distribution, barycenter and stabilometry [31]. Before assessments, according to the manufacturer’s instructions, an initial no-load calibration of the platform was performed. After the subject stepped onto the platform, the virtual sensors were set, corresponding to the 1st and 5th metatarsal heads and the heels in both feet [32]. The subject had to stand in an upright position, barefoot, lower limbs extended, with the feet positioned at an angle of 30° to each other and with 5 cm between the heels [33].

Measurements of plantar pressure and stabilometry were performed for each subject, under the following conditions: no phone (*control*), talking on a smartphone (*talk*) and texting and sending a text message via a smartphone (*text*). The study has a within-subjects design, as each subject completed all three conditions. Each measurement had a duration of 20 s, with a 1-minute period of rest between the testing conditions. During the first testing condition (*control*), the subjects stood on the platform, looking straight ahead, arms along their sides, without talking or moving. The subjects were not using their phones or other devices in this condition. In the following two test conditions, a smartphone (78 × 159 mm) was used. In both the *talk* and the *text* conditions, a phone conversation was simulated: the subjects were interviewed (orally by phone or by text message) by a member of the research group, with the question: “Hello! What plans do you have for the weekend?”. The subjects were instructed not to use abbreviations in their text messages. The researcher and the subjects were placed in different rooms during the two test conditions. For the *talk* assessment, the smartphone was held in the right hand, while for the *text* assessment the smartphone was held with both hands, at the sternum level. The testing was considered invalid and was repeated if the subjects lifted the forefoot or the heel, fell out of position, moved the head, or talked under the *control* or *text* conditions.

The plantar pressure was recorded on the 1st and 5th metatarsal heads and heel, in both the right and left foot; percentage of body weight distribution was calculated by the software for each anatomical region [34]. The ideal load distribution of a subject for the right and the left foot is 16.7% of total weight on 5th metatarsal head, 33.33% of total weight on 1st metatarsal head and 50% of total weight on calcaneus [30,31].

The assessed center of pressure (CoP) for each subject was compared with the theoretical one and average distances from the ideal barycenter were provided by the software for the latero-lateral (CoP_X_) and the anterior-posterior (CoP_Y_) axes [31,35]. A positive value represents a right deviation on the latero-lateral axis and an anterior deviation on the anterior-posterior axis. The absolute mean CoP displacement from the ideal position was calculated based on these deviations [36]. In addition to these parameters, the CoP path length, the 90% confidence ellipse area and the maximum CoP speed were also measured, as recommended by Nagymate and Kiss [37]. The CoP path length represents the length of the subject’s center of gravity shift during the test, measured in millimeters. The confidence ellipse area represents the area (mm^2^) of the ellipse in which all the center of gravity points measured are included and transferred onto a system of Cartesian axes with a confidence level of 90%. The maximum CoP speed represents the average maximum speed of the center of gravity shifting; it is measured in millimeters per second.

In order to quantify the subjects’ dual-tasking ability, the dual-task costs (DTC) were calculated for the stabilometric data (CoP path length, 90% confidence ellipse area and maximum CoP speed) and expressed as a percentage of single-task performance, according to the formula: DTC (%) = (dual-task value–single-task value)*100/ single-task value [38] (single task = *control*; dual-task = *talk*, *text*). A positive DTC value represents a decrease in the ability to maintain postural stability under dual-task conditions [39,40].

### 2.3. Statistical Analysis

Statistical analysis was performed using MedCalc version 8.11 (MedCalc Software bvba, Ostend, Belgium). All data were tested for normality with the Shapiro−Wilk test. The data are presented as mean and standard deviation for normal distributed data and as median and interquartile range for non-normally distributed data. To find the differences in weight distribution and stabilometric data among the three conditions, repeated measures ANOVA with Bonfferroni post hoc analysis and Friedman tests were performed. Wilcoxon tests were performed to compare the DTC between conditions (*talk* vs. *text*). The signed rank sum test was used to compare the DTC with zero reference. Statistical significance was set *p* < 0.05 for all tests.

## 3. Results

The body weight distribution for all three testing conditions is presented in Table 2. A significant main effect was found for the weight load on the left 1st metatarsal head (F_2,68_=3.57, *p =* 0.03), with a greater load on the left 1st metatarsal head in the *talk* compared to the *text* condition (*p =* 0.0006). A significantly lower load on the left heel was observed in the *talk* condition compared with the *text* (*p =* 0.03).

The stabilometric data are presented in Table 3. The main significant effect of the conditions was on the CoP path length (F_2,68_ = 78.67, *p <* 0.001), 90% confidence ellipse area (F_2,68_=30.765, *p <* 0.0001) and maximum CoP speed (F_2,68_ = 50.64, *p <* 0.001). The CoP path length was significantly greater under the *talk* and *text* conditions when compared to the *control* (*p <* 0.05). This parameter was also higher in the *talk* than in the *text* condition (*p <* 0.05). A significantly increased 90% confidence ellipse area was found in *talk* compared to that in *control* (*p =* 0.001) or in *text* (*p =* 0.003). The maximum CoP speed was significantly higher in the *talk* condition when compared to the *control* (*p <* 0.05) and also in the *talk* compared to the *text* condition (*p <* 0.05).

The DTC for CoP path length, 90% confidence ellipse area and maximum CoP speed were all significantly higher in the *talk* compared to *text* condition (*p <* 0.0001) (Table 4). DTCs were reliable and different from zero only for CoP path length and maximum CoP speed in both *talk* (Z = −5.11, *p <* 0.0001 and Z = −5.09, *p <* 0.0001, respectively) and *text* (Z = −3.83, *p =* 0.0001 and Z = −3.24, *p =* 0.001, respectively) conditions and for 90% confidence ellipse area in the *talk* condition (Z = −4.88, *p <* 0.0001).

## 4. Discussion

Using a within-subjects design, we found that talking and texting on a smartphone had a significant effect on postural stability. Thus, our first hypothesis has been validated.

The current data showed that the stabilometric parameters (CoP path length, 90% confidence ellipse area and maximum CoP speed) were significantly affected when using different smartphone functions. The CoP path length and maximum CoP speed were significantly higher in the *talk* and *text* conditions when compared to *control*. This suggests that young adults engaged in a phone conversation or in writing a text message on a smartphone have difficulty in maintaining standing balance. No significant changes between conditions were recorded for the CoP displacement or for the position of the CoP related to the ideal barycenter in anterior-posterior and latero-lateral axes. Previous studies showed that stabilometric analysis performed on a force platform has a good intra- and inter-session reliability [41,42]. The stabilometric parameters are valid measures of postural stability; the smaller their values are, the better the postural stability is [43,44,45]. Laatar et al. also found altered postural control parameters (increased CoP area and CoP displacements on both X and Y axis) while using different cell phone functions like talking or dialing in both young and older subjects [10]. Using different stabilometric parameters (the overall stability index), Rebold et al. analyzed the effects of different cell phone functions on postural stability in 45 college students and also noticed that individuals using their cell phones for texting or talking had worse overall stability index scores compared to control [4].

In addition to previous research, we examined the effect of the second task (talking or texting on a smartphone) on postural balance by calculating the relative difference in the value of stabilometric parameters between the single- and dual-task conditions. Our results showed positive DTC values for CoP path length, 90% confidence area and maximum CoP speed. These results indicate a decrease in the ability to maintain postural stability while talking and texting. In our study talking on the phone proved to be more challenging, with a significant increase in CoP path length, 90% confidence area and maximum CoP speed, compared to texting and control. These findings show that healthy young adults have more difficulty maintaining standing postural balance while talking on the phone, compared to texting, rejecting our second hypothesis that texting would be more demanding. The DTC values were also significantly higher while talking, compared to those achieved when texting. Our results are in accordance with those of Laatar et al., who noticed a significant larger CoP area during phone conversation compared to dialing in both young and older adults [10]. Hsiao et al. also found a poorer postural performance during the verbal dual-task than texting on a smartphone in both young and older participants, possibly due to visual fixation, since texting involves a stable visual fixation [46]. Using a wearable accelerometer, Johnson et al. reported that when they were talking, subjects (aged 18–27 years) exhibited an increased postural sway as compared to standing or texting [47]. In agreement with our results, their data also revealed that talking caused more significant changes in healthy young adults’ ability to maintain their postural balance than texting. It should be noted that Johnson et al. did not use a phone during the talking condition; the subjects, with their arms crossed over their chest, were talking with the investigator standing in front of them about their favorite music. Our results were opposed to those of Rebold et al. who reported a higher overall postural stability index in texting compared to talking on the phone [4]. In their study, texting required more attention than talking or listening to music, thus significantly affecting the postural balance.

In our study we also assessed the static plantar pressure distribution on the 1st and 5th metatarsal heads and calcaneus, of the right and the left foot, in different conditions, namely, no phone, talking on a smartphone, or texting and sending a text message via a smartphone. Talking on a smartphone influenced the weight distribution on the left foot, with an increased pressure on the first metatarsal head and a lower pressure on the heel as compared with message texting. No significant changes were observed in smartphone use conditions when compared to the control. To the best of our knowledge, the impact of talking and texting on smartphones on static plantar pressure in healthy young adults has not been assessed in previous studies. Jin et al. reported that staring at the mobile phone increased the average pressure on the first and second metatarsals compared with normal standing [48].

Since no significant changes were observed in weight distribution between right and left foot among the three conditions, with no significant changes of the CoP positions on the anterior-posterior and latero-lateral axes, the decrease in postural stability control could be due to the cognitive interference or attentional demand of performing a secondary task. Young individuals seem to divide their attention between the two tasks (maintaining postural control and talking/texting on a smartphone), at the cost of affected postural stability. With increasing time being spent talking and texting on smartphones because of the demands of modern lifestyles and of professionally related tasks, the assessment of its effect on the active population is of importance in the field of public health. Due to its consequences for postural stability, recommendations should be made for smartphone use, regarding the amount of time spent talking and texting, the necessity of breaks and compensatory mechanisms related to postural muscle strengthening.

Our study has some limitations: the short testing time (20 s/conditions) did not induce visual or muscular fatigue or any postural changes (e.g., flexed posture); the performance of the cognitive task (texting) was not assessed. The study sample was composed of healthy young subjects that may limit the generalization of the findings to an older population. The impact of using smartphones in the general population could represent a public health problem for modern society [49]. The fact that weight distribution was measured at three anatomical points for each foot may also be a limitation for our study. We intend to continue our research with additional evaluation devices for plantar pressure distribution.

## 5. Conclusions

Postural stability was significantly affected by talking and texting on a smartphone in healthy young adults. They had more difficulty maintaining standing postural balance while talking on the phone than when texting. Talking on the phone also influenced the weight distribution in the left foot at the first metatarsal head and heel as compared with message texting. Future studies are needed to evaluate the effect of the use of smartphones on other daily activities.

## Figures and Tables

**Table 1:** Subjects’ demographic characteristics.

Variables	
Age, years	21.37 ± 1.11
Gender	
Male, *n* (%)	13 (37.14%)
Female, *n* (%)	22 (62.86%)
Weight, kg	63.89 ± 11.08
Height, cm	169.51 ± 8.37
BMI, kg/m^2^	22.13 ± 2.79

Data are presented as mean ± standard deviation; BMI: body mass index.

**Table 2:** Static plantar pressure load distribution.

Variables	Control	Talk	Text	*p*
Right foot (%)	48.98 ± 3.23	49.03 ± 5.26	49 ± 4.81	NS
Right MT1 (%)	17.20 ± 9.03	17.01 ± 9.46	16.14 ± 9.29	NS
Right MT5 (%)	37.35 ± 8.33	38.76 ± 8.87	37.54 ± 8.39	NS
Right heel (%)	45.44 ± 11.27	44.23 ± 12.88	46.32 ± 12.01	NS
Left foot (%)	51.03 ± 3.23	50.97 ± 5.26	51 ± 4.81	NS
Left MT1 (%)	22.69 ± 6.57	22.77 ± 7.65	21.08 ± 7.81	0.03
Left MT5 (%)	28.05 ± 8.33	29.78 ± 7.69	28.67 ± 7.57	NS
Left heel (%)	49.26 ± 9.62	47.45 ± 9.57	50.26 ± 9.23	0.03

Data are presented as mean ± standard deviation; MT1: 1st metatarsal head; MT5: 5th metatarsal head; NS: not significant.

**Table 3:** Stabilometric data across the assessed conditions.

	Control	Talk	Text	*p*
CoP_X_	0 [−3.75–6]	2 [−3–8.75]	1 [−4.5–6]	NS
CoP_Y_	0 [−8.5–10]	1 [−6–5]	−3 [−8–4.5]	NS
CoP displacement (mm)	12.72 [6.33–18.6]	12.37 [6.51–22.01]	12 [8.19–18.38]	NS
CoP path length (mm)	236 [211–258]	317 [283–420.25]	259 [239.25–284]	<0.0001
90% confidence ellipse area (mm^2^)	37 [25.5–51]	115 [54.5–182.25]	58 [20.5–73.25]	<0.0001
Maximum CoP speed (mm/s)	47 [42–51.5]	91 [58.5–117.25]	56 [47.25–66.75]	<0.0001

Data are presented as median [interquartile range]; CoP: center of pressure; NS: not significant.

**Table 4:** Proportional DTC for stabilometric parameters in *talk* and *text* conditions.

	Talk	Text	*p*
DTC CoP displacement (%)	14.31 [−15.56–46.23]	4.62 [−31.84–44.88]	NS
DTC CoP path length (%)	45.58 [29.63–69.48]	10.73 [1.46–24.89]	<0.0001
DTC 90% confidence ellipse area (%)	216.67 [42.69–607.35]	18.18 [−32.14–124.58]	<0.0001
DTC maximum CoP speed (%)	68.08 [39.63–129.14]	17.78 [0.36–40.20]	<0.0001

DTC: dual-task costs. Data are presented as median [interquartile range]; CoP: center of pressure; NS: not significant.

## References

[1] Pellecchia G.L. (2003). Postural sway increases with attentional demands of concurrent cognitive task. Gait Posture.

[2] Huxhold O., Li S.-C., Schmiedek F., Lindenberger U. (2006). Dual-tasking postural control: Aging and the effects of cognitive demand in conjunction with focus of attention. Brain Res. Bull..

[3] Andersson G., Yardley L., Luxon L. (1998). A dual-task study of interference between mental activity and control of balance. Am. J. Otol..

[4] Rebold M.J., Croall C.A., Cumberledge E.A., Sheehan T.P., Dirlam M.T. (2017). The impact of different cell phone functions and their effects on postural stability. Perform. Enhanc. Health.

[5] Hunter M.C., Hoffman M.A. (2001). Postural control: Visual and cognitive manipulations. Gait Posture.

[6] Polskaia N., Richer N., Dionne E., Lajoie Y. (2015). Continuous cognitive task promotes greater postural stability than an internal or external focus of attention. Gait Posture.

[7] Andersson G., Hagman J., Talianzadeh R., Svedberg A., Larsen H.C. (2002). Effect of cognitive load on postural control. Brain Res. Bull..

[8] Maylor E.A., Wing A.M. (1996). Age Differences in Postural Stability are Increased by Additional Cognitive Demands. J. Gerontol. Ser. B.

[9] Woollacott M., Shumway-Cook A. (2002). Attention and the control of posture and gait: A review of an emerging area of research. Gait Posture.

[10] Laatar R., Kachouri H., Borji R., Rebai H., Sahli S. (2017). The effect of cell phone use on postural balance and mobility in older compared to young adults. Physiol. Behav..

[11] Yardley L., Gardner M., Leadbetter A., Lavie N. (1999). Effect of articulatory and mental tasks on postural control. NeuroReport.

[12] Dault M.C., Yardley L., Frank J.S. (2003). Does articulation contribute to modifications of postural control during dual-task paradigms?. Cogn. Brain Res..

[13] Cho S.-H., Choi M.-H., Goo B.-O. (2014). Effect of Smart Phone Use on Dynamic Postural Balance. J. Phys. Ther. Sci..

[14] Nurwulan N.R., Jiang B.C., Iridiastadi H. (2015). Posture and Texting: Effect on Balance in Young Adults. PLoS ONE.

[15] Kim S.-C., Cho W.-S., Cho S.-H. (2020). Effects of smart phone use on lower limb joint angle and dynamic balance during gait. Work.

[16] Haga S., Sano A., Sekine Y., Sato H., Yamaguchi S., Masuda K. (2015). Effects of using a Smart Phone on Pedestrians’ Attention and Walking. Procedia Manuf..

[17] Di Giulio I., McFadyen B.J., Blanchet S., Reeves N.D., Baltzopoulos V., Maganaris C.N. (2020). Mobile phone use impairs stair gait: A pilot study on young adults. Appl. Ergon..

[18] Simmons S.M., Caird J.K., Ta A., Sterzer F., E Hagel B. (2020). Plight of the distracted pedestrian: A research synthesis and meta-analysis of mobile phone use on crossing behaviour. Inj. Prev..

[19] Schabrun S.M., Hoorn W.V.D., Moorcroft A., Greenland C., Hodges P.W. (2014). Texting and Walking: Strategies for Postural Control and Implications for Safety. PLoS ONE.

[20] Crowley P., Madeleine P., Vuillerme N. (2019). The effects of mobile phone use on walking: A dual task study. BMC Res. Notes.

[21] Strubhar A.J., Peterson M.L., Aschwege J., Ganske J., Kelley J., Schulte H. (2015). The effect of text messaging on reactive balance and the temporal and spatial characteristics of gait. Gait Posture.

[22] Goddard E.C., Remler P.T., Roos R.H., Turchyn R. (2018). The Effect of Texting on Balance and Temporospatial Aspects of Gait. West. Undergrad. Res. J. Health Nat. Sci..

[23] Park J.-H., Kang S.-Y., Lee S.-G., Jeon H.-S. (2017). The effects of smart phone gaming duration on muscle activation and spinal posture: Pilot study. Physiother. Theory Pr..

[24] Cochrane M.E., Tshabalala M.D., Hlatswayo N.C., Modipana R.M., Makibelo P.P., Mashale E.P., Pete L.C. (2019). The short-term effect of smartphone usage on the upper-back postures of university students. Cogent Eng..

[25] Lee S., Han S.-K., Lee D.-H. (2016). The Effects of Posture on Neck Flexion Angle While Using a Smartphone according to Duration. J. Korean Soc. Phys. Med..

[26] Szczygieł E., Piotrowski K., Golec J., Czechowska D., Masłoń A., Bac A., Golec E. (2016). Head position influence on stabilographic variables. Acta Bioeng. Biomech..

[27] Zulkifli S.S., Loh W.P., Ping L.W. (2020). A state-of-the-art review of foot pressure. Foot Ankle Surg..

[28] Roman N., Tirziman E., Sorea D., Miclaus R., Repanovici A., Amaricai E., Rogozea L. (2018). Ethical dilemmas in the interdisciplinary approach to informed consent to patients in physiotherapy services in Romania. Rev. Cercet. Interv. Soc..

[29] Faul F., Erdfelder E.R., Lang A.G., Buchner A. (2007). G*Power 3: A flexible statistical power analysis program for the social, behavioral, and biomedical sciences. Behavior. Behav. Res. Methods.

[30] Kapandji A.I. (2011). The Plantar Vault. The Physiology of the Joints. The Lower Limb.

[31] PoData 2.0. http://www.chinesport.com/catalog/posture-analysis/stabilometric-analysis/03001-podata-2-0.

[32] Toprak M., Alptekin H.K., Turhan D. (2018). Correction to: P-12 Assessment of Symmetrigraph and Global Postural System Results for the Posture Analysis of the Healthy Individuals. Chiropr. Man. Ther..

[33] Scoppa F., Gallamini M., Belloni G., Messina G. (2017). Clinical stabilometry standardization: Feet position in the static stabilometric assessment of postural stability. Acta Medica Mediterr..

[34] Gobbi G., Galli D., Carubbi C., Pelosi A., Lillia M., Gatti R., Queirolo V., Costantino C., Vitale M., Saccavini M. (2013). Assessment of body plantar pressure in elite athletes: An observational study. Sport Sci. Health.

[35] Mancini M., Horak F.B. (2010). The relevance of clinical balance assessment tools to differentiate balance deficits. Eur. J. Phys. Rehabil. Med..

[36] Perinetti G. (2006). Dental occlusion and body posture: No detectable correlation. Gait Posture.

[37] Nagymáté G., Kiss R.M. (2016). Replacing redundant stabilometry parameters with ratio and maximum deviation parameters. Proceedings of the 12th IASTED International Conference on Biomedical Engineering, BioMed 2016.

[38] Doumas M., Smolders C., Krampe R. (2008). Task prioritization in aging: Effects of sensory information on concurrent posture and memory performance. Exp. Brain Res..

[39] Tse C.M., Carpenter M.G., Liu-Ambrose T., E Chisholm A., Lam T. (2017). Attentional requirements of postural control in people with spinal cord injury: The effect of dual task. Spinal Cord.

[40] Schaefer S., Krampe R., Lindenberger U., Baltes P.B. (2008). Age differences between children and young adults in the dynamics of dual-task prioritization: Body (balance) versus mind (memory). Dev. Psychol..

[41] Baldini A., Nota A., Assi V., Ballanti F., Cozza P. (2013). Intersession reliability of a posturo-stabilometric test, using a force platform. J. Electromyogr. Kinesiol..

[42] Ruhe A., Fejer R., Walker B. (2010). The test–retest reliability of centre of pressure measures in bipedal static task conditions—A systematic review of the literature. Gait Posture.

[43] Donath L., Roth R., Zahner L., Faude O. (2012). Testing single and double limb standing balance performance: Comparison of COP path length evaluation between two devices. Gait Posture.

[44] Asseman F., Caron O., Crémieux J. (2004). Is there a transfer of postural ability from specific to unspecific postures in elite gymnasts?. Neurosci. Lett..

[45] Paillard T., Noé F. (2006). Effect of expertise and visual contribution on postural control in soccer. Scand. J. Med. Sci. Sports.

[46] Hsiao D., Belur P., Myers P.S., Earhart G.M., Rawson K.S. (2020). The impact of age, surface characteristics, and dual-tasking on postural sway. Arch. Gerontol. Geriatr..

[47] Johnson A., Etter J., Petrillo C., Chen W., Nuzzolo J., McGinnis R. Wearable Sensors Show That Talking, Not Texting, Impairs Postural Control. Proceedings of the 43rd Annual Northeast Bioengineering Conference: New Jersey Institute of Technology.

[48] Jin Z.X., He H., Ruan B., Guo H., Xiong K. (2017). yu Influence of staring at mobile phone on static balance, plantar pressure gait characteristics and lower limb joints in young men. Chin. J. Tissue Eng. Res..

[49] Drugus D., Repanovici A., Popa D., Tirziman E., Roman N., Rogozea L., Miclaus R. (2017). Social impact of public health care in risk management implementation. Rev. Cercet. Interv. Soc..

